# Subclinical Carotid Atherosclerosis in Patients with Rheumatoid Arthritis at Low Cardiovascular Risk

**DOI:** 10.3390/biomedicines11030974

**Published:** 2023-03-21

**Authors:** Elena V. Gerasimova, Tatiana V. Popkova, Daria A. Gerasimova, Yuliya V. Markina, Tatiana V. Kirichenko

**Affiliations:** 1 V.A. Nasonova Research Institute of Rheumatology, 115522 Moscow, Russia; 2 Department of Organization and Economy of Pharmacy, Institute of Pharmacy, I.M. Sechenov First Moscow State Medical University (Sechenov University), 119991 Moscow, Russia; 3 Petrovsky National Research Center of Surgery, 119991 Moscow, Russia; 4 Chazov National Medical Research Center of Cardiology, 121552 Moscow, Russia

**Keywords:** rheumatoid arthritis, carotid atherosclerosis, cardiovascular risk factors, QRISK3, intima-media thickness, sICAM-1, sVCAM, sCD40L

## Abstract

Objective: To evaluate the rate of subclinical carotid atherosclerosis and clinical significance of immunoinflammatory markers in patients with rheumatoid arthritis (RA) at low cardiovascular risk. Materials and Methods: The study included 275 RA patients and a control group of 100 participants without autoimmune diseases. All study participants were at low cardiovascular risk, calculated by the QRISK3 scale (<20%), and free of cardiovascular disease. Ultrasound examination of carotid arteries was performed to measure cIMT and to detect atherosclerotic plaques (ASP) in carotid arteries. sIСАМ-1, sVСАМ, and sCD40L levels were determined by enzyme immunoassay. Results: Carotid ASP was observed more frequently in RA patients (27%) than in the control group (17%), *p* = 0.03. The frequency of ASP in RA patients did not depend on the disease’s stage or activity. There was a significant correlation between cIMT and age, cardiovascular risk determined by QRISK3, level of total cholesterol, LDL, and blood pressure in RA patients, *p* < 0.05 in all cases. No correlation between cIMT and blood levels of sCD40L, sVCAM, and sICAM was found. In RA patients, a higher concentration of sVCAM was detected in the carotid ASP group compared to the non-atherosclerotic group. sCD40L was associated with cIMT and total cholesterol in the ASP group and with total cholesterol and blood pressure in non-atherosclerotic patients. Conclusions: Subclinical atherosclerotic lesions of the carotid arteries were observed significantly more frequently in RA patients with low cardiovascular risk than in the control group. The results of the study demonstrate the association between cIMT, traditional cardiovascular risk factors, and immunoinflammatory markers in RA patients.

## 1. Introduction

Cardiovascular disease (CVD) is the most prevalent and socially significant comorbidity and one of the main causes of premature mortality in rheumatoid arthritis (RA) [1,2,3,4]. Currently, RA is considered an independent cardiovascular risk factor that increases the risk of developing atherosclerotic CVD by about 50%, including in patients with subclinical or early-stage RA [5]. Timely diagnostics based on risk assessment is an extremely important aspect of the prevention of cardiovascular complications in patients with RA [6,7]. At the same time, the stratification of cardiovascular risk in RA is still a serious problem. Insufficient predictive value of traditional cardiovascular risk factors and risk scales, as well as the high frequency of subclinical atherosclerosis, leads to an underestimation of cardiovascular risk in patients with RA [6,8,9]. The use of ultrasound measurement of the thickness of the intima-media complex of carotid arteries (cIMT) in clinical practice has improved the stratification of cardiovascular risk significantly [10,11]. The accelerated development of atherosclerosis in RA is associated with both traditional cardiovascular risk factors and chronic inflammatory status, which determines the production of various inflammatory mediators. Despite the fact that the molecular mechanisms underlying relationships between RA and the development of the atherosclerotic plaques are not well understood, the pathogenesis of atherosclerosis progression in patients with autoimmune diseases is being actively studied for a better understanding of the inflammation–atherosclerosis axis in this group of patients, since it is one of the most actual questions of modern rheumatology [12]. It is known that the progression of the immunopathological process in RA is accompanied by the development of a wide range of immunoinflammatory reactions, which may have pathophysiological significance in the development of atherosclerosis. The imbalance of cytokines and the accumulation of inflammatory mediators contribute to the development of vascular disorders associated with atherogenesis—endothelial dysfunction, vasoconstriction, lipid and lipoprotein peroxidation, hypercoagulation—and later lead to the formation and destabilization of atherosclerotic plaques and the development of cardiovascular complications [13,14]. It was shown that in the assessment of early atherosclerotic vascular changes, the prognostic significance of soluble CD40 ligand (sCD40L) and adhesion molecules—intercellular adhesion molecule-1 (sICAM-1) and vascular cell adhesion molecule-1 (sVCAM-1)—was significantly higher compared to other biological mediators [15,16]. The crucial role of sCD40L and adhesion molecules in pathogenetic mechanisms of atherosclerosis development has been established in numerous studies [17,18]. Activation of the sCD40L is associated with the expression of adhesion molecules and pro-inflammatory cytokines by key cells of the vascular wall leading to the recruitment of immune cells to the arterial endothelium that is considered a main step in the pathogenesis of atherosclerosis. At the same time, it was demonstrated that RA patients have significantly increased levels of sCD40L, ICAM-1, and VCAM-1 than healthy subjects [19,20]. In this regard, these molecules may be considered important mediators in the development of atherosclerotic lesions in rheumatic patients. The aim of the present study was to examine the traditional cardiovascular risk factors, blood levels of sCD40L, ICAM-1, and VCAM-1, ultrasound measurements of carotid atherosclerosis, and to reveal their association in RA patients at low cardiovascular risk compared to healthy controls.

## 2. Materials and Methods

The study was performed in accordance with the Declaration of Helsinki and approved by the local ethics committee. Informed consent to participate in the study was obtained from each participant. The study included 275 RA patients with a low cardiovascular risk free of CVD and 100 participants without autoimmune diseases of the control group matched by sex, age, traditional cardiovascular risk factors, and rate of cardiovascular risk. RA was diagnosed according to the American College of Rheumatology/European Alliance of Associations for Rheumatology (ACR/EULAR) 2010 criteria [21]. The mean age of study participants was 51 [47; 54] years old, 88% of patients were female, and the RA duration was 124 [34; 225] months. The early stage of RA was determined in 38 (14%) patients, advanced stage in 182 (66%) patients, and late stage in 55 (20%) patients. RA activity was evaluated using several scores: disease activity score 28 (DAS28), Clinical Disease Activity Index (CDAI), and Health assessment questionnaires (HAQ). Most patients (69%) had moderate disease activity: DAS28—4.9 [3.7; 5.9] points, CDAI—18 [12; 29] points, and HAQ—1.25 [0.875; 1.75] points; 81% of patients were seropositive for the rheumatoid factor (RF) and 77% for the antibodies against cyclic citrullinated peptides (ACCP). When included in the study, 64% of RA patients received methotrexate (mean dose 20 [15; 27] mg/week, the average duration 110 [21; 174] months; cumulative doses 8,8 [1,7; 13,9]g), 15%—leflunomide (20 mg/day), 12%—sulfasalazine (2000 mg/day), 42%—glucocorticoids (mean dose 4 [2; 6] mg/day, the average duration 75 [15; 175] months; cumulative doses 9,1 [1,8; 21,0] g), and 34%—non-steroid anti-inflammatory preparations. RA patients and participants of the control group have never been treated with statins. Clinical characteristics of patients with RA are presented in Table 1.

The control group included 100 participants without autoimmune diseases with low cardiovascular risk, comparable in sex (female/male 88/12) and age (mean age 47 [45; 51] years). The frequency of traditional cardiovascular risk factors in RA patients and participants of a control group was comparable as well: arterial hypertension in 48% and 42%, dyslipidemia in 62% and 58%, overweight in 45% and 37%, family history for CVD in 43% and 40%, and smoking in 25% and 23% of RA patients and controls, respectively. The QRISK calculator (QResearch Cardiovascular disease Risk Algorithm, QRISK®3-2018), developed for predicting the 10-year risk of coronary heart disease development and cardiovascular events in general medical practice, was used to calculate cardiovascular risk [22]. The QRISK3 algorithm includes almost all traditional cardiovascular risk factors and several additional factors (rheumatoid arthritis, systemic lupus erythematosus, diabetes mellitus, chronic kidney disease, ethnicity).

The ultrasound examination of the carotid arteries was carried out on the ultrasound system Esaote MyLab Twice (Esaote S.p.A, Genoa, Italy). Atherosclerotic lesions of the carotid arteries were assessed by the detection of atherosclerotic plaques determined as a local increase of intima-media thickness ≥ 1.5 mm. The examination included scanning of the left and right common carotid arteries, the carotid bifurcation area, as well as external and internal carotid arteries with a focus on the far wall of the artery in three fixed projections—anterior, lateral, and posterior. Measurement of cIMT was performed at the far wall of the common carotid artery 10 mm opposite the top of the carotid bifurcation. cIMT was measured as the distance from the leading edge of the first echogenic area to the leading edge of the second echogenic area [23]. One researcher was responsible for all cIMT measurements throughout the study. The whole procedure was recorded on a digital scan medium for subsequent analysis using the dedicated software package M’Ath (Metris, SRL, Villers-Bretonneux, France). The average of three measurements (in the anterior, posterior, and lateral projections) was considered an integral indicator of cIMT. The concentrations of total cholesterol, high-density lipoproteins (HDL), and triglycerides (TG) were determined by standard enzymatic methods, and the level of low-density lipoproteins (LDL) was calculated using the Friedwald formula: LDL-C = cholesterol − TG/5 − HDL cholesterol. The levels of C-reactive protein (CRP) and IgM-RF in blood serum were measured by the immunonephelometric method using a BN Pro Spec analyzer (Siemens, Munich, Germany). The concentration of sVCAM, sICAM, and sCD40L in venous blood serum was determined by enzyme immunoassay using reagent kits and according to Bender MedSystems (Burlingame, CA, USA) protocols.

Statistical data processing was carried out using Statistica 12 and SPSS 14.0 software. Results are presented as the median and interquartile range (Me [25th; 75th percentile]). The χ^2^ test was used to compare the frequencies of qualitative data in groups. The groups were compared using the nonparametric Mann–Whitney test; correlation analysis was performed by Spearman’s rank correlation method. Differences were considered significant at *p* < 0.05.

## 3. Results

Atherosclerotic plaques of the carotid arteries were observed more frequently in RA patients (27%) with low cardiovascular risk than in the control group (17%), *p* = 0.03. Carotid atherosclerosis was detected in 50% of men and 24% of women, *p* < 0.01. The frequency of carotid atherosclerotic plaques in RA patients with low cardiovascular risk did not depend on the stage and activity of the disease. In RA patients, a relationship between cIMT and age (R = 0.48), the value of cardiovascular risk assessed by QRISK3 scale (R = 0.36), the level of total cholesterol (R = 0.28), LDL (R = 0.18), systolic blood pressure (SBP) (R = 0.37), and diastolic blood pressure (DBP) (R = 0.38) was found, *p* < 0.05 in all cases. In the control group, a similar relationship with age (R = 0.41), concentrations of total cholesterol (R = 0.22), LDL (R = 0.26), levels of SBP (R = 0.18), and DBP (R = 0.23) was determined, *p* < 0.05 in all cases. Table 2 presents the comparison of study participants in basic traditional cardiovascular risk factors and carotid atherosclerosis parameters, as well as blood levels of adhesion molecules (sIСАМ-1, sVСАМ) and sCD40L.

When comparing the blood levels of sCD40L, sVCAM, and sICAM of RA patients and control group participants, a higher concentration of sVCAM and a tendency to increase the blood level of sICAM in RA patients were revealed in comparison with the control group. No differences in blood levels of sCD40L were determined between groups. Correlation analysis did not reveal the association between the cIMT and blood concentrations of sICAM-1, sVCAM, and sCD40L both in patients with RA and in the control group. To search for risk factors for the development of carotid atherosclerotic plaques in RA patients with low cardiovascular risk, the method of multivariate logistic regression analysis was used with the calculation of OR and 95% CI. The sensitivity of the model was 51.5%, the specificity was 96.2%, and the coefficient of multiple determination R2 was 0.396. The factors associated with the risk of carotid atherosclerosis are presented in Table 3. Thus, in RA patients with low cardiovascular risk, the presence of arterial hypertension increased the risk of developing carotid atherosclerotic plaques by two times and dyslipidemia by almost three times.

For further analysis, patients with RA and low cardiovascular risk were divided into two groups depending on the presence of atherosclerotic plaques in carotid arteries. The first group consisted of 74 RA patients with carotid atherosclerosis; the second group included 201 RA patients without atherosclerosis. There were more men (21%) and patients with dyslipidemia (88%) in the atherosclerotic group of study participants in comparison with the non-atherosclerotic group (*p* < 0.05). It was found that RA patients with carotid atherosclerotic plaques were older and had higher body mass index (BMI), rate of cardiovascular risk calculated by the QRISK3 scale, as well as serum levels of cholesterol, LDL, triglycerides, and sVCAM concentrations compared to patients without carotid atherosclerotic plaques (Table 4).

Then, we analyzed the correlation between immunoinflammatory markers and traditional cardiovascular risk factors in both groups. It was found that serum level of sCD40L correlated significantly with cIMT (R = 0.40, *p* = 0.04) and total cholesterol blood level (R = 0.38, *p* = 0.01) in a group of patients with atherosclerosis and with total cholesterol levels (R = 0.41, *p* = 0.02) and DBP (R = 0.3, *p* = 0.04) in the non-atherosclerosis group. It was also detected in patients without atherosclerosis that serum levels of sVCAM were associated with RF (R = 0.52, *p* = 0.01), while serum levels of sICAM were associated with DAS28 (R = 0.44, *p* = 0.04).

## 4. Discussion

Stratification of cardiovascular risk in patients with RA remains a difficult problem, especially in cases with low cardiovascular risk. Cardiovascular risk calculated with the use of special scores does not always reflect the real risk of CVD in patients with RA [24,25]. The risk for CVD is 1.5 times higher in RA patients, and 10-year CVD risk scores underestimate the cardiovascular risk of rheumatic patients [24]. The choice of the QRISK3 calculator for risk calculation was based on its advantage in predicting the risk of CVD in patients with autoimmune rheumatic diseases [26,27,28]. Therefore, in the work of A. Corrales et al. [27], the best predictors of CVD and mortality in patients with RA, adjusted for age, sex, and disease duration, were the QRISK3 risk score and the detection of carotid atherosclerotic plaque. Given the low cardiovascular risk in RA patients, the role of surrogate markers increases, which primarily includes the visualization of subclinical atherosclerotic vascular lesions [27,29]. The high prevalence of subclinical atherosclerosis in patients with RA compared with subjects without autoimmune diseases is observed already in the early stages of RA and has a similar prevalence in patients with early and late stages of the disease [30,31,32]. In our previous study, carotid atherosclerosis (atherosclerotic plaques in carotid arteries) was diagnosed in 55.4% of patients with early untreated RA mean aged 56 years old, as well as increased mean cIMT over 0.9 mm was detected in 51.4% of RA patients [33]. According to other authors, subclinical carotid atherosclerosis was found in 21–46% of patients with RA [34,35,36]. The progression of atherosclerosis in carotid arteries was observed 3–5-fold more in patients with RA than in individuals without autoimmune diseases [35,36]. In the current study, carotid atherosclerosis was detected in 27% of RA patients with low cardiovascular risk. The data obtained are consistent with the work of S. Hannawi et al. [35], who demonstrated that manifestations of asymptomatic carotid atherosclerosis were observed more often in RA patients with low cardiovascular risk than in subjects of a comparable control group (21% vs. 4%, *p* = 0.01). The association of immune disorder due to autoimmune processes and traditional cardiovascular risk factors, primarily dyslipidemia and hypertension, play an important role in the accelerated development of atherosclerosis in RA. The current study demonstrates the dependence of the cIMT on traditional risk factors for CVD, such as gender, age, serum levels of lipids, and blood pressure in patients with RA, which matches the results of other studies [33,35,36].

It has been shown that chronic systemic inflammation plays an important role in accelerated atherogenesis in autoimmune diseases. Inflammation can have a direct impact on the development of atherosclerosis or indirectly enhance the effect of traditional cardiovascular risk factors [14,31,32]. One of the mechanisms of the atherosclerotic process connecting inflammation and atherothrombosis is the activation of the CD40/CD40L signaling system. CD40L is a transmembrane glycoprotein belonging to the tumor necrosis factor (TNF) family. CD40 and CD40L are expressed by various cells, including the cells of atherosclerotic plaque: B-lymphocytes, monocytes/macrophages, and endothelial and smooth muscle cells. Increased expression of sCD40L is considered an important factor in the immunopathogenesis of RA. The mechanism of its action is realized through the polyclonal activation of B-lymphocytes, the formation of autoantibodies, the activation of endothelial cells, and the increased production of pro-inflammatory cytokines [37,38]. The diagnostic and prognostic value of sCD40L in patients with CVD and healthy individuals is discussed in the literature. The Ludwigshafen Risk and Cardiovascular Health study demonstrates that sCD40L independently predicted an increase in cardiovascular risk in the general population; a significant association between elevated sCD40L plasma levels and short-term all-cause and cardiovascular mortality was observed in 2759 study participants [39]. The association of elevated blood concentrations of sCD40L with several cardiovascular risk factors has been demonstrated in clinical trials. In particular, it was shown previously that there is a relationship between the CD40/CD40L system and cholesterol metabolism in patients with moderate hypercholesterolemia [40]. Our data indicate a similar relationship between sCD40L levels and cholesterol levels in RA patients with low cardiovascular risk. In addition, blood pressure can affect the level of sCD40L, which was confirmed in the current work and matches the results of other studies [41]. The increased blood level of sCD40L was previously demonstrated in RA patients compared to the control group, but in that study, no association of sCD40L with disease activity and markers of inflammation such as erythrocyte sedimentation rate and C-reactive protein was revealed. There are few works that assess the clinical significance of pCD40L as a marker of atherosclerotic vascular lesions in RA and SLE [42]. Our study found a positive correlation of this marker with traditional and non-traditional risk factors, as well as with atherosclerotic lesions of the carotid arteries in RA patients with low cardiovascular risk. The relationship between sCD40L level and cIMT in RA patients with atherosclerosis identified in our study may have clinical and diagnostic significance in the prediction of subclinical atherosclerosis in RA patients. Activation of the CD40/CD40L system is considered a key step in the development of atherosclerosis and its complications and is associated with the synthesis of adhesion molecules and chemokines by monocytes/macrophages, endotheliocytes, platelet-leukocyte adhesion, increased expression of tissue factor and matrix metalloproteinases, and activation and proliferation of vascular smooth muscle cells [15,43,44]. The binding of CD40L to the receptor on endotheliocytes and smooth muscle cells induces the expression of leukocyte adhesion molecules such as VCAM, E-selectin, and ICAM that causes the recruitment of immune-inflammatory cells with the subsequent development of local inflammation, leading to the formation of atherosclerotic lesions [45,46]. In RA, adhesion molecules VCAM and ICAM produced by synovial fibroblasts play an important role in supporting inflammation, promoting recruiting and adhesion of circulating inflammatory cells to endothelium and, consequently, their extravasation at the site of inflammation and tissue damage [47,48,49]. It has been shown in our study that patients with RA have a higher concentration of sVCAM compared to the individual without autoimmune diseases, and the level of sVCAM and sICAM in the peripheral blood is associated with immunological markers (RF) and RA activity indicators (DAS28), respectively. Similar data have been obtained in several other studies demonstrating elevated levels of adhesion molecules in the blood of RA patients compared to healthy controls and their association with DAS28 and RF [48,50]. sICAM-1 and sVCAM-1 are well-known molecules involved in the pathogenesis of atherosclerotic plaques [51]. In the work of JF.Varona et al., sICAM-1 and sVCAM-1 were strongly associated with early atherosclerotic disease in patients with low/intermediate cardiovascular risk [52]. sICAM-1 and sVCAM-1 contribute to the development of atherosclerosis through different mechanisms, and what mechanism is leading is not yet fully understood. On the one hand, the binding of these soluble adhesion molecules to circulating leukocyte receptors before they come into contact with the vessel wall has an anti-adhesive potential that may limit the immune-inflammatory response [53]. On the other hand, the response of macrophages to sICAM-1 lead to the production of MIP-2 and TNF-a through an NFkappaB-dependent mechanism, which in turn enhances inflammation [54].

## 5. Conclusions

Thus, cIMT is considered to be the most informative marker for the identification of the risk of CVD development in patients with RA. In our study, subclinical atherosclerotic lesions of the carotid arteries were detected much more often in RA patients with low cardiovascular risk than in the control group and were found in a quarter of patients. Therefore, ultrasound scanning of carotid arteries should be recommended for RA patients with low risk of CVD as a routine examination in general clinical practice. The relationship between the cIMT with traditional cardiovascular risk factors (age, sex, blood lipid levels, blood pressure) as well as with immunoinflammatory markers of cardiovascular risk (sCD40L, sVCAM-1) in patients with RA has been demonstrated. Serum levels of sCD40L and sVCAM-1 in RA patients appear to be useful biomarkers for detecting early subclinical atherosclerotic lesions in RA patients with low cardiovascular risk.

## Figures and Tables

**Table 1:** Clinical characteristics of study participants with RA.

Indicator	RA Patients (*n* = 275)
Age, years	51 [47; 54]
Sex, female/male, *n* (%)	243 (88)/32 (12)
RA duration, months	124 [34; 225]
Stage of disease: early, *n* (%) advanced, *n* (%) late, *n* (%)	38 (14) 182 (66) 55 (20)
Extra-articular manifestations, *n* (%)	127 (46)
DAS28, points	4.9 [3.7; 5.9]
RF +, %	81
ACCP +, %	77
CDAI, points	18 [12; 29]
HAQ	1.25 [0.875; 1.75]
Basic anti-inflammatory therapy, % Methotrexate, % Leflunomide, % Sulfasalazine, %	90 64 15 12
Glucocorticoids, %	42
Non-steroid anti-inflammatory preparations, %	34

**Table 2:** Comparison of traditional cardiovascular risk factors, atherosclerosis measurements, blood levels of sCD40L, and adhesion molecules in RA and control patients.

	RA Group (*n* = 275)	Control Group (*n* = 100)	*p*
ASP, *n* (%)	47 (27)	17 (17)	0.03
cIMT mean, mm	0.73 [0.60; 0.81]	0.73 [0.69; 0.79]	n.s.
cIMT max, mm	0.87 [0.75; 1.01]	0,86 [0.78; 1.00]	n.s.
Total cholesterol >5.0 mmol/L, *n* (%)	108 (62)	58 (58)	n.s.
Total cholesterol, mmol/l	5.7 [4.9; 6.4]	5.7 [5.2; 6.5]	n.s.
LDL >2.6 mmol/L, *n* (%)	99 (57)	49 (49)	n.s.
LDL, mmol/L	3.9 [2.6; 4.6]	3.7 [2.3; 4.6]	n.s.
HDL <1.0 mmol/L in men or <1.2 mmol/L in women, *n* (%)	79 (45)	10 (10)	0.01
HDL, mmol/L	1.3 [1.0; 1.6]	1,5 [1.2; 1.8]	<0.01
Triglycerides >1.7 mmol/L, *n* (%)	27 (15)	8 (8)	n.s.
Triglycerides, mmol/L	0.9 [0.5; 1.2]	0.7 [0.4; 1.1]	n.s.
Atherogenic index	3.4 [1.9; 3.8]	2.8 [2.2; 3.0]	0.01
sCD40L, ng/mL	6.5 [1.7; 11.8]	5.6 [2.0; 9.6]	n.s.
sVCAM, ng/mL	1520 [945; 1915]	790 [605; 1307]	0.01
sIСАМ, ng/mL	332 [274; 405]	285 [247; 344]	0.06

n.s.—not significant.

**Table 3:** Factors associated with the development of carotid atherosclerotic plaques in RA patients with low cardiovascular risk.

	OR	95% CI	*p*
Arterial hypertension	2.26	1.15–4.56	0.035
Dislipidemia	2.91	1.35–6.40	0.005
Body mass index > 30 kg/m^2^	2.07	0.91–4.76	0.09
Male gender	0.54	0.060–4.34	0.62
Smoking	1.50	0.65–3.45	0.35
Age > 50 years	1.76	0.84–3.69	0.12
Menopause	2.23	0.99–4.93	0.05

**Table 4:** Cardiovascular risk factors in atherosclerotic and non-atherosclerotic patients with RA.

	Atherosclerotic Group (*n* = 74)	Non-Atherosclerotic Group (*n* = 201)	*p*
Sex, female/male, *n* (%)	58 (79)/15 (21)	181 (92)/15 (8)	0.04
Age, years	54 [50; 56]	50 [46; 53]	<0.001
Body mass index, kg/m^2^	25 [22; 27]	22 [19; 26]	0.01
QRISK 3, points	7.0 [5.9; 15.1]	6.2 [4.0; 10.9]	0.03
Dyslipidemia, *n* (%)	65 (88)	189 (60)	0.03
Total cholesterol, mmol/L	6.2 [5.7; 7.2]	5.7 [4.9; 6.4]	0.01
LDL, mmol/L	4.3 [3.8; 5.1]	4.0 [3.4; 4.7]	0.03
HDL, mmol/L	1.3 [1.0; 1.5]	1.3 [1.1; 1.5]	n.s.
Triglycerides, mmol/L	0.9 [0.5; 1.4]	0.7 [0.5; 1.1]	0.02
sVCAM, ng/mL	1680 [1460; 3350]	1210 [865; 1810]	0.01

n.s.—not significant.

## Data Availability

Data is available from the corresponding author upon request.

## References

[1] Pappas D.A., Nyberg F., Kremer J.M., Lampl K., Reed G.W., Horne L., Ho M., Onofrei A., Malaviya A.N., Rillo O.L. (2018). Prevalence of Cardiovascular Disease and Major Risk Factors in Patients with Rheumatoid Arthritis: A Multinational Cross-Sectional Study. Clin. Rheumatol..

[2] Balsa A., Lojo-Oliveira L., Alperi-López M., García-Manrique M., Ordóñez-Cañizares C., Pérez L., Ruiz-Esquide V., Corrales A., Narváez J., Rey-Rey J. (2019). Prevalence of Comorbidities in Rheumatoid Arthritis and Evaluation of Their Monitoring in Clinical Practice: The Spanish Cohort of the COMORA Study. Reum. Clin..

[3] Wang H., Li X., Gong G. (2020). Cardiovascular Outcomes in Patients with Co-Existing Coronary Artery Disease and Rheumatoid Arthritis: A Systematic Review and Meta-Analysis. Medicine.

[4] Lee Y.K., Ahn G.Y., Lee J., Shin J.M., Lee T.H., Park D.J., Song Y.J., Kim M.K., Bae S.C. (2021). Excess Mortality Persists in Patients with Rheumatoid Arthritis. Int. J. Rheum. Dis..

[5] Hansildaar R., Vedder D., Baniaamam M., Tausche A.K., Gerritsen M., Nurmohamed M.T. (2021). Cardiovascular Risk in Inflammatory Arthritis: Rheumatoid Arthritis and Gout. Lancet Rheumatol..

[6] Conroy R.M., Pyörälä K., Fitzgerald A.P., Sans S., Menotti A., De Backer G., De Bacquer D., Ducimetière P., Jousilahti P., Keil U. (2003). Estimation of Ten-Year Risk of Fatal Cardiovascular Disease in Europe: The SCORE Project. Eur. Heart J..

[7] Agca R., Heslinga S.C., Rollefstad S., Heslinga M., McInnes I.B., Peters M.J.L., Kvien T.K., Dougados M., Radner H., Atzeni F. (2017). EULAR Recommendations for Cardiovascular Disease Risk Management in Patients with Rheumatoid Arthritis and Other Forms of Inflammatory Joint Disorders: 2015/2016 Update. Ann. Rheum. Dis..

[8] Dessein P.H., Semb A.G. (2013). Could Cardiovascular Disease Risk Stratification and Management in Rheumatoid Arthritis Be Enhanced?. Ann. Rheum. Dis..

[9] Colaco K., Ocampo V., Ayala A.P., Harvey P., Gladman D.D., Piguet V., Eder L. (2020). Predictive Utility of Cardiovascular Risk Prediction Algorithms in Inflammatory Rheumatic Diseases: A Systematic Review. J. Rheumatol..

[10] O’Leary D.H., Bots M.L. (2010). Imaging of Atherosclerosis: Carotid Intima-Media Thickness. Eur. Heart J..

[11] Khanna N.N., Jamthikar A.D., Gupta D., Nicolaides A., Araki T., Saba L., Cuadrado-Godia E., Sharma A., Omerzu T., Suri H.S. (2019). Performance Evaluation of 10-Year Ultrasound Image-Based Stroke/Cardiovascular (CV) Risk Calculator by Comparing against Ten Conventional CV Risk Calculators: A Diabetic Study. Comput. Biol. Med..

[12] Popescu D., Rezus E., Codruta Badescu M., Dima N., Nicoleta P., Isac S., Dragoi I.-T., Rezus C. (2023). Cardiovascular Risk Assessment in Rheumatoid Arthritis: Accelerated Atherosclerosis, New Biomarkers, and the Effects of Biological Therapy. Life.

[13] Reiss A.B., Silverman A., Khalfan M., Vernice N.A., Kasselman L.J., Carsons S.E., De Leon J. (2019). Accelerated Atherosclerosis in Rheumatoid Arthritis: Mechanisms and Treatment. Curr. Pharm. Des..

[14] Fomicheva O.A., Popkova T.V., Krougly L.B., Gerasimova E.V., Novikova D.S., Pogorelova O.A. (2021). Factors of Progression and Occurrence of Atherosclerosis in Rheumatoid Arthritis. Kardiologiia.

[15] Lacy M., Bürger C., Shami A., Ahmadsei M., Winkels H., Nitz K., van Tiel C.M., Seijkens T.T.P., Kusters P.J.H., Karshovka E. (2021). Cell-Specific and Divergent Roles of the CD40L-CD40 Axis in Atherosclerotic Vascular Disease. Nat. Commun..

[16] Pereira-Da-silva T., Napoleão P., Pinheiro T., Selas M., Silva F., Ferreira R.C., Carmo M.M. (2021). The Proinflammatory Soluble CD40 Ligand Is Associated with the Systemic Extent of Stable Atherosclerosis. Medicina.

[17] Galkina E., Ley K. (2007). Vascular Adhesion Molecules in Atherosclerosis. Arter. Thromb. Vasc. Biol..

[18] Pereira-da-Silva T., Ferreira V., Castelo A., Caldeira D., Napoleão P., Pinheiro T., Ferreira R.C., Carmo M.M. (2021). Soluble CD40 Ligand Expression in Stable Atherosclerosis: A Systematic Review and Meta-Analysis. Atherosclerosis.

[19] Södergren A., Karp K., Bengtsson C., Möller B., Rantapää-Dahlqvist S., Wållberg-Jonsson S. (2019). Biomarkers Associated with Cardiovascular Disease in Patients with Early Rheumatoid Arthritis. PLoS ONE.

[20] Román-Fernández I.V., García-Chagollán M., Cerpa-Cruz S., Jave-Suárez L.F., Palafox-Sánchez C.A., García-Arellano S., Sánchez-Zuno G.A., Muñoz-Valle J.F. (2019). Assessment of CD40 and CD40L Expression in Rheumatoid Arthritis Patients, Association with Clinical Features and DAS28. Clin. Exp. Med..

[21] Kay J., Upchurch K.S. (2012). ACR/EULAR 2010 Rheumatoid Arthritis Classification Criteria. Rheumatology.

[22] Solomon D.H., Greenberg J., Curtis J.R., Liu M., Farkouh M.E., Tsao P., Kremer J.M., Etzel C.J. (2015). Derivation and Internal Validation of an Expanded Cardiovascular Risk Prediction Score for Rheumatoid Arthritis: A Consortium of Rheumatology Researchers of North America Registry Study. Arthritis Rheumatol..

[23] Touboul P.J., Grobbee D.E., Den Ruijter H. (2012). Assessment of Subclinical Atherosclerosis by Carotid Intima Media Thickness: Technical Issues. Eur. J. Prev. Cardiol..

[24] Mackey R.H., Kuller L.H., Moreland L.W. (2017). Cardiovascular Disease Risk in Patients with Rheumatic Diseases. Clin. Geriatr. Med..

[25] Gerasimova E.V., Popkova T.V., Gerasimova D.A., Glukhova S.I., Nasonov E.L., Lila A.M. (2021). Application of Cardiovascular Risk Scales to Identify Carotid Atherosclerosis in Patients with Rheumatoid Arthritis. Ter. Arkh..

[26] Edwards N., Langford-Smith A.W.W., Parker B.J., Bruce I.N., Reynolds J.A., Alexander M.Y., McCarthy E.M., Wilkinson F.L. (2018). QRISK3 Improves Detection of Cardiovascular Disease Risk in Patients with Systemic Lupus Erythematosus. Lupus Sci. Med..

[27] Corrales A., Vegas-Revenga N., Rueda-Gotor J., Portilla V., Atienza-Mateo B., Blanco R., Castañeda S., Ferraz-Amaro I., Llorca J., González-Gay M.A. (2020). Carotid Plaques as Predictors of Cardiovascular Events in Patients with Rheumatoid Arthritis. Results from a 5-Year-Prospective Follow-up Study. Semin. Arthritis Rheum..

[28] Di Battista M., Barsotti S., Della Rossa A., Mosca M. (2022). Cardiovascular Burden in Systemic Sclerosis: QRISK3 versus Framingham for Risk Estimation. Mod. Rheumatol..

[29] Kobayashi H., Giles J.T., Polak J.F., Blumenthal R.S., Leffell M.S., Szklo M., Petri M., Gelber A.C., Post W., Bathon J.M. (2010). Increased Prevalence of Carotid Artery Atherosclerosis in Rheumatoid Arthritis Is Artery-Specific. J. Rheumatol..

[30] Hannawi S., Haluska B., Marwick T.H., Thomas R. (2007). Atherosclerotic Disease Is Increased in Recent-Onset Rheumatoid Arthritis: A Critical Role for Inflammation. Arthritis Res..

[31] Giles J.T., Post W.S., Blumenthal R.S., Polak J., Petri M., Gelber A.C., Szklo M., Bathon J.M. (2011). Longitudinal Predictors of Progression of Carotid Atherosclerosis in Rheumatoid Arthritis. Arthritis Rheum..

[32] Ambrosino P., Lupoli R., di Minno A., Tasso M., Peluso R., di Minno M.N.D. (2015). Subclinical Atherosclerosis in Patients with Rheumatoid Arthritis. A Meta-Analysis of Literature Studies. Thromb. Haemost..

[33] Udachkina E.V., Novikova D.S., Popkova T.V., Kirillova I.G., Markelova E.I., Gorbunova Y.N., Karateev D.E., Luchikhina E.L., Demidova N.V., Borisova M.A. (2018). Progression of Carotid Artery Atherosclerosis during Treatment to Target in Patients with Early Rheumatoid Arthritis. Rheumatol. Sci. Pract..

[34] Dehghan P., Rajaei A., Moeineddin R., Alizadeh A.M. (2015). Prevalence of Atherosclerosis in Patients with Inactive Rheumatoid Arthritis. Clin. Rheumatol..

[35] Hannawi S.M.A., Hannawi H., Alokaily F., Salmi I. (2020). Al Subclinical Atherosclerosis in Rheumatoid Arthritis Patients of the Gulf Cooperated Council. Saudi Med. J..

[36] Dalbeni A., Giollo A., Bevilacqua M., Cioffi G., Tagetti A., Cattazzo F., Orsolini G., Ognibeni F., Minuz P., Rossini M. (2020). Traditional Cardiovascular Risk Factors and Residual Disease Activity Are Associated with Atherosclerosis Progression in Rheumatoid Arthritis Patients. Hypertens. Res..

[37] Liu M.F., Chao S.C., Wang C.R., Lei H.Y. (2001). Expression of CD40 and CD40 Ligand among Cell Populations within Rheumatoid Synovial Compartment. Autoimmunity.

[38] Alaaeddine N., Hassan G.S., Yacoub D., Mourad W. (2012). CD154: An Immunoinflammatory Mediator in Systemic Lupus Erythematosus and Rheumatoid Arthritis. Clin. Dev. Immunol..

[39] Gergei I., Kälsch T., Scharnagl H., Kleber M.E., Zirlik A., März W., Krämer B.K., Kälsch A.I. (2019). Association of Soluble CD40L with Short-Term and Long-Term Cardiovascular and All-Cause Mortality: The Ludwigshafen Risk and Cardiovascular Health (LURIC) Study. Atherosclerosis.

[40] Garlichs C.D., John S., Schmeier A., Eskafi S., Stumpf C., Karl M., Goppelt-Struebe M., Schmieder R., Daniel W.G. (2001). Upregulation of CD40 and CD40 Ligand (CD154) in Patients with Moderate Hypercholesterolemia. Circulation.

[41] Guzel M., Dogru M.T., Simsek V., Demir V., Alp C., Kandemir H., Yildirim N., Kisa U. (2019). Influence of Circadian Blood Pressure Alterations on Serum SCUBE-1 and Soluble CD40 Ligand Levels in Patients with Essential Hypertension. Am. J. Cardiovasc. Dis..

[42] Pamuk O.N., Tozkir H., Uyanik M.S., Gurkan H., Saritas F., Duymaz J., Donmez S., Yazar M., Pamuk G.E. (2014). PECAM-1 Gene Polymorphisms and Soluble PECAM-1 Level in Rheumatoid Arthritis and Systemic Lupus Erythematosus Patients: Any Link with Clinical Atherosclerotic Events?. Clin. Rheumatol..

[43] Schönbeck U., Sukhova G.K., Shimizu K., Mach F., Libby P. (2000). Inhibition of CD40 Signaling Limits Evolution of Established Atherosclerosis in Mice. Proc. Natl. Acad. Sci. USA.

[44] Gocmen A.Y., Ocak G.A., Ozbilim G., Delibas N., Gumuslu S. (2013). Effect of Atorvastatin on Atherosclerotic Plaque Formation and Platelet Activation in Hypercholesterolemic Rats. Can. J. Physiol. Pharm..

[45] Hakonarson H., Kim C., Whelan R., Campbell D., Grunstein M.M. (2001). Bi-Directional Activation between Human Airway Smooth Muscle Cells and T Lymphocytes: Role in Induction of Altered Airway Responsiveness. J. Immunol..

[46] Antoniades C., Bakogiannis C., Tousoulis D., Antonopoulos A.S., Stefanadis C. (2009). The CD40/CD40 Ligand System: Linking Inflammation with Atherothrombosis. J. Am. Coll. Cardiol..

[47] Vascular Cell Adhesion Molecule 1 (CD106): A Multifaceted Regulator of Joint Inflammation. https://onlinelibrary.wiley.com/doi/epdf/10.1002/1529-0131%28200105%2944%3A5%3C985%3A%3AAID-ANR176%3E3.0.CO%3B2-P.

[48] Wang L., Ding Y., Guo X., Zhao Q. (2015). Role and Mechanism of Vascular Cell Adhesion Molecule-1 in the Development of Rheumatoid Arthritis. Exp. Med..

[49] Liu Y., Wei W., Hong C., Wang Y., Sun X., Ma J., Zheng F. (2019). Calreticulin Induced Endothelial ICAM-1 up-Regulation Associated with Tristetraprolin Expression Alteration through PI3K/Akt/ENOS/P38 MAPK Signaling Pathway in Rheumatoid Arthritis. Mol. Immunol..

[50] Navarro-Hernández R.E., Oregon-Romero E., Del Mercado M.V., Rangel-Villalobos H., Palafox-Sánchez C.A., Muoz-Valle J.F. (2009). Expression of ICAM1 and VCAM1 Serum Levels in Rheumatoid Arthritis Clinical Activity. Association with Genetic Polymorphisms. Dis. Mrk..

[51] Gómez Rosso L., Benítez M.B., Fornari M.C., Berardi V., Lynch S., Schreier L., Wikinski R., Cuniberti L., Brites F. (2008). Alterations in Cell Adhesion Molecules and Other Biomarkers of Cardiovascular Disease in Patients with Metabolic Syndrome. Atherosclerosis.

[52] Varona J.F., Ortiz-Regalón R., Sánchez-Vera I., López-Melgar B., García-Durango C., Castellano Vázquez J.M., Solís J., Fernández-Friera L., Vidal-Vanaclocha F. (2019). Soluble ICAM 1 and VCAM 1 Blood Levels Alert on Subclinical Atherosclerosis in Non Smokers with Asymptomatic Metabolic Syndrome. Arch. Med. Res..

[53] Videm V., Albrigtsen M. (2008). Soluble ICAM-1 and VCAM-1 as Markers of Endothelial Activation. Scand. J. Immunol..

[54] Soluble ICAM-1 Activates Lung Macrophages and Enhances Lung Injury—PubMed. https://pubmed.ncbi.nlm.nih.gov/9759893/.

